# Musculoskeletal problems and health perceptions of women who applied to the orthopedic clinic after cesarean section

**DOI:** 10.1590/1806-9282.20241804

**Published:** 2025-07-07

**Authors:** Muhsin Dursun

**Affiliations:** 1 Adana Ortadogu Special Hospital, Department of Orthopedics and Traumatology – Adana, Turkey.

**Keywords:** Pain, Musculoskeletal diseases, Subjective health, Cesarean section

## Abstract

**OBJECTIVE:**

The aim of this study was to retrospectively investigate the musculoskeletal problems and health perceptions of women who applied to the orthopedic clinic after cesarean delivery.

**METHODS:**

This study used a descriptive and cross-sectional design. It was conducted with 243 women who sought treatment in an orthopedic clinic between January 2023 and May 2024. Women who agreed to participate in the study, who had a cesarean section for the first time, who did not have any musculoskeletal problems before the cesarean section, who had musculoskeletal problems for a year or more after the cesarean section, and who sought treatment in the orthopedics and traumatology outpatient clinic were included in the study. Data were collected through the "Personal Information Form," the "Extended Nordic Musculoskeletal Questionnaire," and the "Nottingham Health Profile."

**RESULTS:**

The average age of the participating women was 32.61±6.89 years. Of all the women, 44.8% experienced pain mostly in the back and lower back region within the last year following cesarean delivery. Nottingham Health Profile scores were negatively affected by several variables such as age, weight gained during pregnancy, BMI, and musculoskeletal disorders (p<0.050). An analysis of the effect sizes showed that Cohen's d values for back and lower back regions according to pain experience within the last year were 1.07 and 1.07, respectively, indicating a high effect size.

**CONCLUSIONS:**

Musculoskeletal problems, particularly in the back and lower back regions, negatively affect women's overall health perception after cesarean delivery.

## INTRODUCTION

The World Health Organization (WHO) defines musculoskeletal disorders as health problems involving muscles, tendons, the skeleton, cartilage, ligaments, and nerves. Musculoskeletal disorders include all types of mild and temporary complaints, including irreversible and incapacitating injuries^1^.

Women physiologically experience several hormonal and anatomical changes during the pregnancy period. These physiological adaptations could also negatively affect the musculoskeletal system^2^ and may lead to chronic musculoskeletal problems that may persist throughout life in some pregnant women^2,3^. Cesarean section is a major obstetric surgical procedure that has an increasing rate worldwide. A recent report published by WHO states that cesarean delivery continues to increase worldwide, accounting for 21% of all childbirths. With an estimated 29% of all childbirths by cesarean section by 2030, this rate is expected to continue to increase in the next decade^1^. Musculoskeletal problems could be experienced following cesarean section due to fascia and bone injuries, relaxation of spinal muscles, flattening of lumbar lordosis, and stretching of lumbosacral ligaments and joint capsules^4,5^.

Health perception is defined as an individual's assessment of his/her own health status^6^. The health problems could negatively affect the health perception. Positive health perception is highly important for individuals to acquire healthy lifestyle behaviors^7^. On the other hand, the literature includes findings indicating that musculoskeletal problems negatively affect health perception^7–9^. However, no studies in the literature were found to have investigated the effect of musculoskeletal problems on health perception following cesarean section.

Investigation of the musculoskeletal problems that may be experienced following cesarean section and determination of the level of the effect of these complaints on health perception is believed to guide the prevention of the negative effects of postpartum musculoskeletal system problems and help women who have undergone cesarean section take preventive measures. Ignoring these problems could disrupt diagnostic processes and often lead to missed preventive and therapeutic opportunities. The purpose of this study is to examine the musculoskeletal problems experienced by women after cesarean delivery and assess the impact of these issues on their overall health perception. By investigating these factors, the study aims to raise awareness about the potential long-term effects of cesarean section on musculoskeletal health and provide insight into strategies for early detection, prevention, and treatment to improve the quality of life for women in the postpartum period.

## METHODS

### Study design and participants

**Figure 1:**
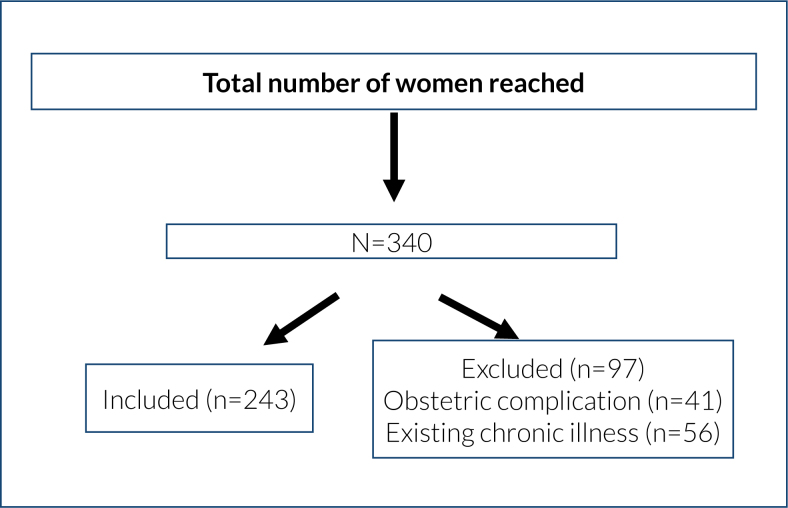
Participant flow chart.

This study used a retrospective and cross-sectional design and was conducted with women who sought treatment in the orthopedic clinic of a hospital located in Adana between January 2023 and May 2024. The sample size was calculated at a 95% confidence level using the "G. Power-3.1.9.2" program. No studies in the literature were found to have analyzed the relationship between musculoskeletal problems and health perception following cesarean section. For this reason, a power calculation was performed using correlation analysis based on the medium effect size for sample calculation. Taking alpha 0.05 and beta (power) value 0.90, the minimum sample size was calculated as 243^10,11^. A total of 243 participants were included in the study (Figure 1).

**Inclusion criteria:** Women who voluntarily participated, were open to communication, spoke Turkish, had their first cesarean section, had no prior musculoskeletal issues, developed musculoskeletal problems persisting for a year post-cesarean, and sought treatment in the orthopedics and traumatology outpatient clinic.

**Exclusion criteria:** Women with vertigo, two or more cesarean sections, psychiatric disorders, or a history of surgical intervention other than childbirth.

### Data collection

Data were collected through the "Personal Information Form," the "Extended Nordic Musculoskeletal Questionnaire," and the "Nottingham Health Profile." Data collection was completed in 5–10 min.

### Personal information form

The Personal Information Form was developed by the researchers in line with the literature and included 10 questions concerning sociodemographic and obstetric characteristics of women and their spouses/partners^3,7^.

### Extended Nordic Musculoskeletal Questionnaire

The Extended Nordic Musculoskeletal Questionnaire was utilized to investigate musculoskeletal problems. The scale was developed by Kuorinka et al.^12^, and its Turkish validity and reliability were performed by Genç et al.^13^. The questionnaire assesses whether the respondent has experienced a problem (pain, soreness, discomfort) in the musculoskeletal system (neck, shoulders, back, elbows, wrists/hands, lower back, hips/thighs, knees, ankles/feet) at any time within the last 12 months and whether there has been pain within the last seven days. In the answers given to the questions, zero points are given for a "No" answer, and one point is given for a "Yes" answer. The test has no cut-off limit, and statistical analyses cannot be performed on the total scores^13^.

### Nottingham Health Profile

The Nottingham Health Profile was used to measure individuals’ health perception. The NHP was developed by Doll et al.^14^, and its Turkish validity and reliability were performed by Kücükdeveci et al.^6^. The scale consists of 38 questions with six different areas, which include physical mobility, pain, sleep, energy, social isolation, and emotional reactions. Items are responded to as yes/no. Each question has a different score weight, and each domain is scored between 0 and 100. Higher scores obtained from the scale indicate a worse health perception. Each domain in the scale is calculated separately. In the calculation process, the number of questions answered yes in each sub-group is divided by the total number of questions in the same sub-group, and the result is multiplied by 100^6^.

### Statistical analysis

The data obtained in the study were analyzed using SPSS (Statistical Package for Social Sciences) for the Windows 25.0 program. Data analysis included descriptive statistics (numbers, percentages, means, standard deviations). The comparison of quantitative data in normally distributed data was performed using the independent t-test for the difference between two independent groups and one-way ANOVA (F test) for more than two independent groups. The effect size was calculated with Cohen's d. Statistical significance was assessed at a significance level of p<0.05.

### Ethics statement

Ethics committee approval was obtained from Ortadoğu Hospital (dated 01/02/2023 Decision No: 953). The study was conducted following the principles of the Declaration of Helsinki. Data were collected by the researcher by meeting the participants face-to-face after they were informed about data collection, voluntary participation, and confidentiality issues, and their verbal and written consents were obtained.

## RESULTS

Table 1 presents the participants’ sociodemographic, obstetric, and musculoskeletal characteristics. The average age of the participating women was found to be 32.61±6.89 years.

**Table 1:** Comparison of the participants’ health perception scores by their sociodemographic characteristics.

Variables		n	%	NHP			
				X	SD	Test value	p-value
Age	Below 33	122	49.2	105.58	98.50	**-6.242** t	**0.000** *
	33 years and over	126	50.8	213.79	166.87		
Weight gained during pregnancy	Less than 13 kg	103	41.5	140.32	138.67	**2.542** t	**0.012** *
	13 kg and over	145	58.5	189.05	155.57		
BMI	Less than 18.50	34	13.7	79.08	78.63	**27.736** F	**0.000** *
	18.51–24.90	135	54.4	116.53	118.23		
	24.91–29.90	45	18.1	241.35	139.96		
	29.91 and over	34	13,7	309.97	165.27		
Occupation	Private sector	53	21.4	98.92	89.47	**9.173** F	**0.000** *
	Public employee	25	10.1	119.79	125.21		
	Self-employment	42	16.9	116.92	104.68		
	Housewife	117	47.2	217.04	169.84		
	Other	11	4.4	116.09	86.32		
Education level	Primary school	24	9.7	340.00	160.41	**19.784** F	**0.000** *
	Secondary school	56	22.6	216.70	168.16		
	High school	92	37.1	127.14	113.22		
	University and above	76	30.6	102.99	100.86		
Number of pregnancies	1	86	34.7	98.82	91.36	**34.755** F	**0.000** *
	2	88	35.5	131.28	113.08		
	3	43	17.3	197.57	173.09		
	4	31	12.5	363.65	136.58		
Having exercises during pregnancy (Pilates, etc.)	Yes	58	23.4	80.19	89.24	**-6.495** t	**0.000** *
	No	190	76.6	185.10	153.23		
Walking 30 ms a day during pregnancy	Yes	117	47.2	111.80	109.95	**-5.277** t	**0.000** *
	No	131	52.8	204.11	162.94		
Musculoskeletal problems beginning during pregnancy	Yes	65	26.2	134.39	127.79	**4.246** t	**0.000** *
	No	183	73.8	234.26	173.67		
Type of anesthesia used during cesarean section	Spinal anesthesia	138	55.6	141.42	139.12	**7.752** F	**0.004** *
	Epidural anesthesia	6	2.4	80.79	57.71		
	General anesthesia	104	41.9	190.56	156.72		

t independent groups t-test;

F One-way ANOVA (F test)

* p<0.05.

BMI: body mass index; NHP: Nottingham Health Profile; SD: standard deviation. Significant values are written in bold.

It was determined that 44.8% of the women experienced pain in the back and lower back within the last year following cesarean section (Table 2).

**Table 2:** Prevalence of musculoskeletal pain after cesarean section and health perception mean scores.

ENMQ			Pain experienced within the last year		Restriction of daily life activities (at home or out of home) due to pain within the last year		Pain experienced within the last seven days	
			N	%	n	%	n	%
Neck		Yes	112	45.2	92	37.1	75	30.2
		No	136	54.8	156	62.9	173	69.8
Shoulders		Yes	101	40.7	87	35.1	79	31.9
		No	147	59.3	161	64.9	169	68.1
Elbows		Yes	78	31.5	62	25.0	55	22.2
		No	170	68.5	186	75.0	193	77.8
Wrists/hands		Yes	67	27.0	52	21.0	41	16.5
		No	181	73.0	196	79.0	207	83.5
Back		Yes	111	44.8	106	42.7	87	35.1
		No	137	55.2	142	57.3	161	64.9
Lower back		Yes	111	44.8	118	47.6	90	36.3
		No	137	55.2	130	52.4	158	63.7
Hips/thighs		Yes	92	37.1	97	39.1	79	31.9
		No	156	62.9	151	60.9	169	68.1
Knees		Yes	95	38.3	95	38.3	73	29.4
		No	153	61.7	153	61.7	175	70.6
Ankle/feet		Yes	85	34.3	72	29.0	62	25.0
		No	163	65.7	176	71.0	186	75.0
NHP and subscales		χ±SD				Min–max		
	Pain	54.45±28.17				0–100		
	Emotional reactions	22.31±25.63				0–100		
	Sleep	47.34±33.22				0–100		
	Social isolation	23.23±29.50				0–100		
	Physical activity	71.17±26.32				0–100		
	Energy	42.07±41.12				0–100		
	NHP total	260.56±147.59				0–600		

ENMQ: Extended Nordic Musculoskeletal Questionnaire; NHP: Nottingham Health Profile; SD: standard deviation.

The participants’ total NHP score was found to be 260.56±147.59, and the highest score was found in the physical activity (71.17±26.32) sub-scale (Table 3).

**Table 3:** Comparison of the health perception mean scores according to the prevalence of musculoskeletal pain participants’ pain after cesarean section.

Pain region		Nottingham Health Profile						
			n	χ	SD	t	-test p-value	Cohen d
Pain experienced within the last year	Neck	Yes	112	215.24	166.28	**5.612**	**0.000** *	**0.71**
		No	136	115.53	112.24			
	Shoulders	Yes	111	223.90	168.51	**6.277**	**0.000** *	**0.84**
		No	137	109.24	103.29			
	Elbows	Yes	111	216.52	168.11	**5.471**	**0.000** *	**0.72**
		No	137	115.22	109.90			
	Wrists/eller	Yes	67	223.49	167.63	**3.794**	**0.000** *	**0.60**
		No	181	137.27	132.55			
	Back	Yes	78	257.86	173.77	**6.647**	**0.000** *	**1.07**
		No	170	115.92	108.19			
	Lower back	Yes	101	243.82	169.47	**7.549**	**0.000** *	**1.07**
		No	147	103.35	95.34			
	Hips/thighs	Yes	92	223.90	175.01	**4.937**	**0.000** *	**0.72**
		No	156	123.21	113.77			
	Knees	Yes	95	240.25	176.08	**6.538**	**0.000** *	**0.96**
		No	153	111.08	98.95			
	Ankle/feet	Yes	85	230.02	173.98	**5.039**	**0.000** *	**0.76**
		No	163	124.34	116.90			
Pain experienced within the last 7 days	Neck	Yes	75	229.90	173.94	**4.483**	**0.000** *	**0.70**
		No	173	130.50	123.53			
	Shoulders	Yes	87	221.19	170.58	**4.520**	**0.000** *	**0.66**
		No	161	127.80	122.10			
	Elbows	Yes	90	224.73	175.00	**4.892**	**0.000** *	**0.72**
		No	158	124.01	114.92			
	Wrists/eller	Yes	41	234.23	178.68	**2.994**	**0.004** *	**0.61**
		No	207	145.97	136.47			
	Back	Yes	79	241.00	175.84	**5.447**	**0.000** *	**0.86**
		No	169	122.96	114.92			
	Lower back	Yes	55	248.69	171.09	**4.548**	**0.000** *	**0.80**
		No	193	135.45	130.12			
	Hips/thighs	Yes	79	226.53	166.34	**4.584**	**0.000** *	**0.68**
		No	169	129.72	127.19			
	Knees	Yes	73	223.89	170.58	**4.042**	**0.000** *	**0.63**
		No	175	134.15	128.45			
	Ankle/feet	Yes	62	213.50	164.90	**3.037**	**0.003** *	**0.48**
		No	186	142.92	137.35			

* p<0.05; independent sample t-test was used. SD: standard deviation. Significant values are written in bold.

A statistically significant difference was found between the participants’ NHP total mean score and their age, weight gain during pregnancy, daily walking for 30 min and exercise during pregnancy, and persistent musculoskeletal problems beginning during pregnancy (p<0.050). Further analysis results showed a statistically significant difference between the NHP total mean score and the variables of BMI, occupation, education, number of pregnancies, and type of anesthesia (p<0.050) (Table 1).

Women's NHP scores were negatively affected by their pain experience within the last year (p<0.050). An analysis of the effect sizes showed that Cohen's d values for the back and lower back regions according to the pain experience within the last year were 1.07 and 1.07, respectively (Table 3). According to these values, back and lower back regions negatively affected women's health perception with a high effect size^15^.

## DISCUSSION

According to the study results, among women experiencing back and lower back pain following cesarean section, these pains were found to significantly negatively affect health perception. Additionally, the overall NHP mean score was significantly associated with factors such as age, weight gain during pregnancy, daily walking, exercise during pregnancy, BMI, occupation, education, number of pregnancies, and type of anesthesia. The effect size analysis revealed that back and lower back pain had a high negative impact on women's health perception (Cohen's d≈1.07). These findings demonstrate that musculoskeletal problems following cesarean section play a notable role in influencing women's overall health perception.

Pregnancy-related postpartum musculoskeletal pain may be experienced^16^, yet because the cesarean section is a major surgical operation, these pains persist for a long time^17^, which may lead to sequelae. Therefore, women are mostly referred to orthopedics and traumatology specialists for the treatment of musculoskeletal pain experienced for a long time following cesarean section. The present study is the first study that investigated the effect of musculoskeletal problems following cesarean section on health perception and the affecting factors. Around half of the women who participated in the study were found to experience back and lower back pain within the last year. It was reported that 50% of women who experienced lower back pain during pregnancy continued to experience this pain for one year after delivery^18^. Smith et al. found that a significant reduction in abdominal muscle strength after cesarean section increased the risk of chronic lower back pain^19^. This study found that women's health perception was negatively affected. Jones and Brown reported that, in addition to postoperative pain, patients who underwent cesarean section experienced temporary limitations in mobility, which may be associated with long-term musculoskeletal problems^20^. Women are expected to return to their daily life activities 4–6 weeks after delivery^19,21^. This study found that women had difficulty in performing their daily life activities because they mostly experienced back and lower back pain, which increased their health perception score.

Postpartum health perception was found to be negatively affected by the increase in age and weight gain during pregnancy. Musculoskeletal system changes characterized by decreased joint mobility and slower movement time are encountered with an increase in age and excessive weight gain^16^. The women in this study were 33 years of age or older and gained 13 kg or more weight during pregnancy, which might have shortened joint movements and movement time and decreased health perception. Being obese and being a housewife also decreased women's health perception in this study. Zerf^22^ reported a positive relationship between being an obese housewife and weakness of the musculoskeletal system. Problems experienced in the musculoskeletal system are considered to negatively affect health perception^22^. In our study, women who walked and exercised during pregnancy had higher postpartum health perceptions. In their systematic analysis, Liu et al.^23^ found that women who performed combined exercise and yoga during pregnancy and who had a high level of physical activity improved postpartum health perception.

In this study, the health perception was negatively affected in women who had musculoskeletal problems during pregnancy. Diagnostic processes may be disrupted when musculoskeletal pain during pregnancy is neglected^24^. Missing preventive and therapeutic opportunities may negatively affect postpartum health perception. In our study, the health perception was low in women who had their fourth pregnancy. Musculoskeletal changes occurring during pregnancy are physiologic adaptations necessary for a successful pregnancy and delivery. However, as the number of pregnancies increases, these physiologic adaptations may negatively affect the musculoskeletal system, which might have caused a decrease in the health perception in our study. The health perception was found to be low in women who underwent cesarean section with general anesthesia in our study. Women may experience back and lower back pain following cesarean section due to the relaxation of the spinal muscles after anesthesia^5^. A retrospective cohort study conducted on women reported no relationship between the type of anesthesia and lower back pain^25^. The different results may be related to the differences in the sample groups.

Our study determined that it negatively affects women's self-perceived health due to the complaint of back and lower back pain the most in all body regions within the last year. This decrease was found to have a high effect size^15^. Postpartum lower back pain may be related to biomechanical and physiologic changes occurring in the musculoskeletal system during pregnancy. In women, low back pain causes restriction of activities of daily living^20^. Thus, musculoskeletal problems also negatively affect health perception.

### Limitations of the study

This study was conducted with women admitted to only one hospital, and the results cannot be generalized to the population. The lack of similar studies in the literature and the presentation of factors affecting health perception with effect sizes are the strengths of the study.

## CONCLUSION

In this study, it was demonstrated that musculoskeletal pain following cesarean section, especially back and lower back pain, significantly and adversely affected women's health perception. Nearly half of the participants experienced such pain within 1 year post-delivery, with many reporting persistent symptoms stemming from pregnancy. Moreover, increased age, excessive weight gain, obesity, and low levels of physical activity were associated with poorer health perception, while exercising during pregnancy yielded positive outcomes. The findings also indicate that surgical factors such as a reduction in abdominal muscle strength and temporary mobility limitations may contribute to chronic pain and functional impairments. Overall, these results underscore the critical need for effective interventions and rehabilitative strategies to mitigate the long-term musculoskeletal effects of cesarean section and enhance women's overall well-being.

## Data Availability

The datasets generated and/or analyzed during the current study are available from the corresponding author upon reasonable request.
